# Horizontal and vertical integrative analysis methods for mental disorders omics data

**DOI:** 10.1038/s41598-019-49718-5

**Published:** 2019-09-17

**Authors:** Shuaichao Wang, Xingjie Shi, Mengyun Wu, Shuangge Ma

**Affiliations:** 1 SJTU-Yale Joint Center for Biostatistics, Department of Bioinformatics and Biostatistics, School of Life Sciences and Biotechnology, Shanghai Jiao Tong University, Shanghai, 200240 China; 2 School of Economics, Nanjing University of Finance and Economics, Nanjing, 210046 China; 3 School of Statistics and Management, Shanghai University of Finance and Economics, Shanghai, 200433 China; 4 Department of Biostatistics, Yale University, New Haven, CT 06520 USA

**Keywords:** Genetics, Molecular medicine

## Abstract

In recent biomedical studies, omics profiling has been extensively conducted on various types of mental disorders. In most of the existing analyses, a single type of mental disorder and a single type of omics measurement are analyzed. In the study of other complex diseases, integrative analysis, both vertical and horizontal integration, has been conducted and shown to bring significantly new insights into disease etiology, progression, biomarkers, and treatment. In this article, we showcase the applicability of integrative analysis to mental disorders. In particular, the horizontal integration of bipolar disorder and schizophrenia and the vertical integration of gene expression and copy number variation data are conducted. The analysis is based on the sparse principal component analysis, penalization, and other advanced statistical techniques. In data analysis, integration leads to biologically sensible findings, including the disease-related gene expressions, copy number variations, and their associations, which differ from the “benchmark” analysis. Overall, this study suggests the potential of integrative analysis in mental disorder research.

## Introduction

Mental disorders have been posing an increasing public health concern. Two types of mental disorders that are of essential importance are bipolar disorder and schizophrenia, which have been shown to affect about 1% and 0.5% of the population globally^1,2^. Bipolar disorder is a type of brain order and also known as manic-depressive illness. It can cause unusual shifts in mood, energy, and activity levels, and affect the ability to carry out common tasks. Schizophrenia is a chronic and severe mental disorder. It has a direct impact on thinking, feeling, and behaving. People with schizophrenia often seem to have “lost touch with reality”. Although schizophrenia may be less common than some other mental disorders, the symptoms can be more disabling. Extensive studies have been conducted to understand the etiology and progression of bipolar disorder and schizophrenia. For example, childhood trauma has been suggested as associated with bipolar disorder and probably interacted with genetic susceptibility factors^3^. High paternal age and urbanization at birth have been suggested as possible risk factors for schizophrenia^4^.

In the past decades, we have witnessed significant advancements in omics profiling techniques, and studies have been conducted searching for molecular risk factors for the etiology and progression of bipolar disorder, schizophrenia, and other mental disorders^5^. For example, Kordi-Tamandani and Mir^6^ conducted a gene expression study and identified three groups of functionally related genes that are associated with bipolar disorder and schizophrenia and involved in energy metabolism, mitochondrial function, and others. An analysis of copy number variations (CNVs) in a family-based study suggested the importance of CNVs of genes MAGI1 and MAGI2 in the etiology of bipolar disorder and schizophrenia^7^. Epigenetic studies have also been conducted. For example, Abdolmaleky, *et al*.^8^ found hypermethylation of the reelin (RELN) promoter in the brain of schizophrenic patients. In another study, the DNA methylation status of SOX10 was reported to be associated with the development of schizophrenia^9^. Our literature review suggests that most of the existing studies, including the aforementioned ones, analyze a single type of mental disorder and a single type of omics measurement, hence being limited.

In recent biomedical studies on complex diseases, integrative analysis is gaining popularity fast. There are two main families of integration. In *horizontal integration*, data on different (but usually related) diseases are analyzed, whereas in *vertical integration*, multiple types/sources of data on the same disease are analyzed. An example of horizontal integration is Cava, *et al*.^10^, which jointly analyzed gene expression data on sixteen types of cancers. An example of vertical integration is Chen, *et al*.^11^, which proposed a BRIDGE method and integrated multi-omics data, including protein interactions, gene sequences, and gene expressions, to detect disease markers for obesity and type 2 diabetes. Jiang, *et al*.^12^ developed a novel statistical model to integrate gene expressions, CNVs, and methylation and predict the prognosis of melanoma. Examples also include the prostate cancer study in Taylor, *et al*.^13^, breast cancer study in Hendrickx, *et al*.^14^, and others.

The symptoms, and clinical, environmental, and social risk factors of bipolar disorder and schizophrenia are “related”, suggesting the relatedness of the two diseases^15^. In addition, studies have also shown that some common genetic factors may affect the occurrence of both diseases^16^, suggesting their connections at the molecular level. Logotheti, *et al*.^17^ compared the differentially expressed genes of the two diseases (against normal controls) and found that they shared the downregulation of +K and +Na transporting ATPases. Shao and Vawter^18^ identified 78 genes that are significantly dysregulated in both diseases, including AGXT2L1, SLC1A2, and others. *The aforementioned and other published evidences suggest that it can be reasonable to conduct the horizontal integrative analysis of bipolar disorder and schizophrenia*. The existence of regulations among different types of omics measurements, for example regulations of gene expressions by CNVs, is relatively “independent” of diseases. *With the same rationale as for cancer, diabetes, and other complex diseases, it can be of interest to also conduct the vertical integrative analysis for bipolar disorder and schizophrenia*.

In the literature, there are a few related studies. Studies such as Logotheti, *et al*.^17^ analyzed data on different mental disorders separately and then compared results across diseases to identify overlapping findings. Such studies basically take a meta-analysis strategy, which, as shown in recent studies^19^, is not as effective as the integrative analysis strategy. There are also studies, for example Schubert, *et al*.^20^, that confirmed findings in one type of omics measurement by conducting functional analysis of other types of omics measurements. However, there is a lack of integration in the discovery process. In addition, in these studies, the most advanced statistical techniques have not been adopted.

The goal of this study is to showcase conducting both horizontal and vertical integrative analysis of mental disorders. Although the adopted analytic techniques are much related to those in the study of cancer and other complex diseases, this article is the first to comprehensively apply them in mental disorder studies and can valuably serve as a prototype for future studies. Compared to the existing omics analysis of mental disorders, it has significant advancements in analytic techniques. It is noted that although bipolar and schizophrenia, and gene expression and CNV data are analyzed in this article, the methodologies described can be directly applicable to other mental disorders and other types of omics data. The availability of analysis software enables other researchers to conveniently apply these methods. In addition, the findings from integrative analysis may complement those in the literature using “classic” analysis.

## Methods

### Stanley Medical Research Institute mental disorder data

The Stanley Medical Research Institute (SMRI) is one of the largest organizations supporting research on the causes and treatment of bipolar disorder and schizophrenia. Data analyzed in this article are downloaded directly from the SMRI Online Genomics Database website. To get the download access, researchers are first required to register with the web link https://www.stanleygenomics.org/contact.html. Then data are available and can be downloaded freely from https://www.stanleygenomics.org/stanley/studySummary.jsp. This data has been considered in the literature^21^, however, using relatively simple analysis techniques. For 86 subjects, a total of 20,515 gene expression measurements are available, which were measured using the Affy Hgu133A chips. For CNVs, single nucleotide polymorphism (SNP) data measured by Affy SNP5.0 chip are available. CNV measurements are extracted from the SNP data following a standard procedure using the PennCNV software^22^. A total of 22,428 CNV measurements are available on 153 subjects. After data matching, a total of 71 subjects have both gene expression and CNV measurements (on 19,053 genes), including 23 bipolar disorders, 24 schizophrenics, and 24 normal controls.

As suggested in the literature, the number of genes relevant to bipolar disorder and schizophrenia is not expected to be large. Also with consideration on sample size, we conduct a prescreening, which can improve the reliability of analysis and also reduce computational cost. Specifically, we identify genes in three signaling pathways using the Kyoto Encyclopedia of Genes and Genomes (KEGG) database. The first is the ubiquitin mediated proteolysis (UMP) pathway, which contains 133 genes. It has been suggested that the UMP pathway plays an important role in the treatment of damaged and toxic proteins through ubiquitin-dependent protein hydrolysis, and the disorder of the UMP pathway can affect the occurrence of bipolar disorder and schizophrenia^23,24^. The second is the tryptophan metabolism pathway which contains 40 genes. Tryptophan is an important component of 5-serotonin in brain which has been shown to make people feel calm and mild^25^. The imbalance of tryptophan metabolism has been observed in bipolar disorders and schizophrenics^26^. The third is the neurotrophin signaling pathway, which contains 130 genes. Published studies have shown that the brain-derived neurotrophic factor has the functions of preventing neuron death, promoting the development, differentiation, repair and regeneration of neurons, and strengthening the transduction of synaptic signals^27^, and that the neurotrophin signaling pathway is importantly associated with bipolar disorder and schizophrenia^28^.

It is noted that although these three pathways have been previously indicated as highly important for bipolar and schizophrenia, we by no means suggest that they are the most important or a lack of significance of other pathways. They are sufficient for the purpose of showcasing integrative analysis. It is also noted that this prescreening is not essential. As the sample size is limited in our analysis, we conduct prescreening to reduce dimension for improving estimation stability, as well as reducing computational cost. Other prescreening measures, such as p-value based on marginal regression, can also be used. We adopt this prior information-based approach to increase interpretability. This strategy has also been commonly adopted in published studies^29,30^. If a larger sample size become available, the analysis of all genes can be conducted directly and may lead to more definitive and comprehensive findings.

### Data

For the *k*th type of samples (bipolar disorder, schizophrenia, or normal), assume *n*^(*k*)^ independent subjects with $({{\boldsymbol{x}}}_{i}^{(k)},{{\boldsymbol{z}}}_{i}^{(k)},{y}_{i}^{(k)})$ for *i* = 1, …, *n*^(*k*)^, where for the *i*th subject, ${{\boldsymbol{x}}}_{i}^{(k)}=({x}_{i1}^{(k)},\mathrm{...},{x}_{ip}^{(k)})$ and ${{\boldsymbol{z}}}_{i}^{(k)}=({z}_{i1}^{(k)},\mathrm{...},{z}_{ip}^{(k)})$ are the *p*-dimensional vectors of gene expression and CNV measurements, and ${y}_{i}^{(k)}\in \{0,1\}$ is the binary response with ${y}_{i}^{(k)}=0$ for a normal subject and 1 for bipolar disorder or schizophrenia. The SMRI data has a case-control design, and the outcome variable is binary. Integrative analysis described below can also be conducted on other types of designs/outcome variables. To simplify notation, we have used the same dimension for gene expressions and CNVs and note that in analysis there is no requirement on the matching of gene expressions and their regulators. Integrative analysis can be conducted from multiple different perspectives. Below we describe three types of analysis, which are perhaps more popular in the literature.

### Vertical integrative analysis of multi-omics data

**Figure 1:**
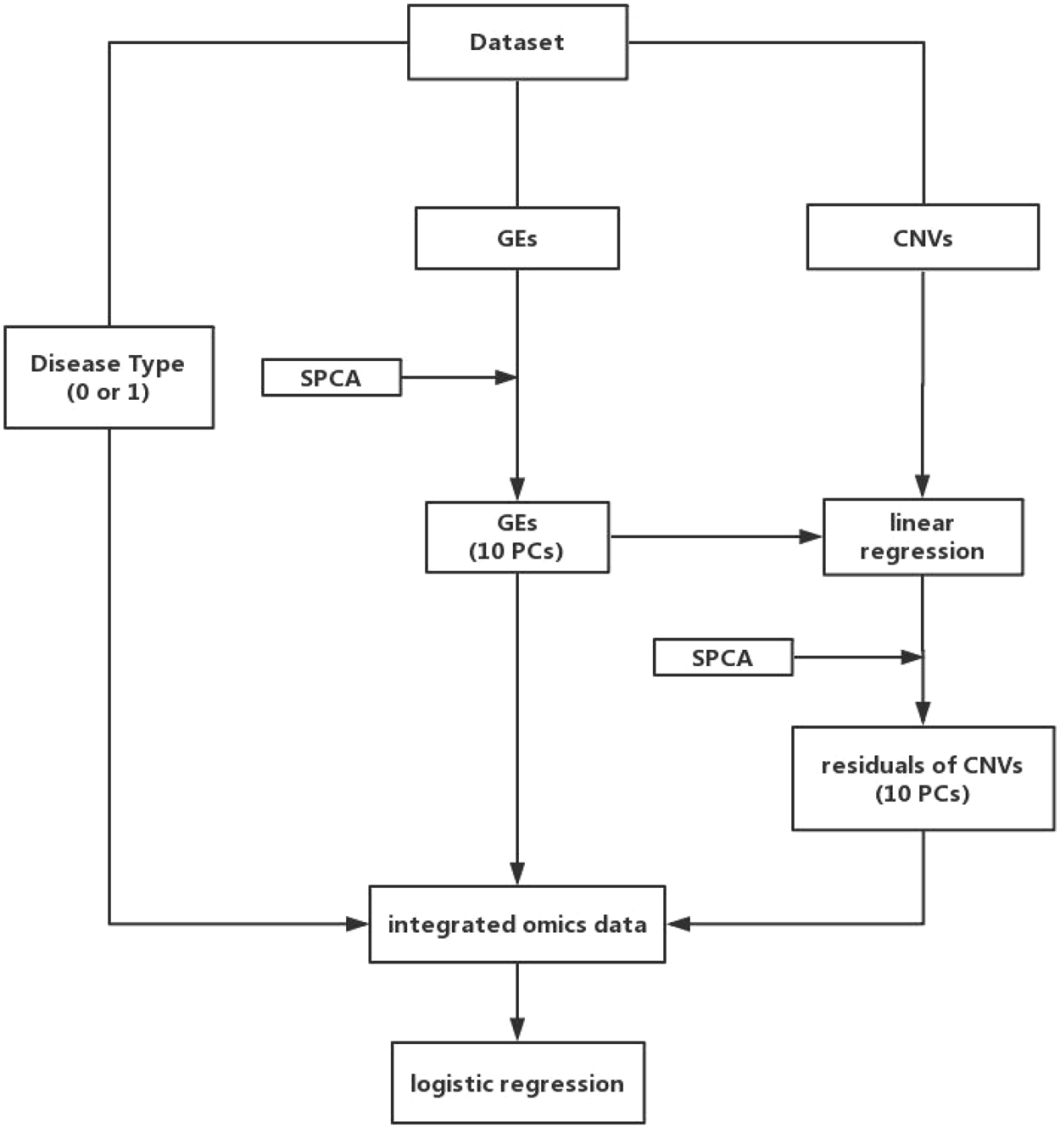
Flowchart of vertical integrative analysis.

In multiple published studies, analysis has been conducted building risk models using omics data. In this set of integrative analysis, the goal is to build a more comprehensive model using multiple types of omics measurements (in this particular case, gene expression and CNV). The overall flowchart of analysis is provided in Fig. 1. The analysis is built on the SPCA (sparse principal component analysis) and other techniques. It can effectively accommodate the regulations between different types of omics measurements, which, if not properly accounted for, can lead to co-linearity in model building. Accommodating the regulations can also make the analysis more interpretable.

The analysis (referred to as A1) proceeds as follows. In the first step, for each type of disease samples separately, we apply SPCA to gene expressions for reducing dimension and accommodating high correlations among genes^31^. The top ten sparse PCs with the largest variances are selected to represent the effects of all gene expressions and used for downstream analysis. Denote them as ${{\boldsymbol{x}}}_{pc,i}^{(k)}=({x}_{pc,i1}^{(k)},\ldots ,{x}_{pc,i10}^{(k)})$, $i=1,\ldots ,\,{n}^{(k)}$.

In the second step, we consider the CNV-gene expression regression model

$${{\boldsymbol{z}}}_{i}^{(k)}={{\boldsymbol{x}}}_{pc,i}^{(k)}{{\boldsymbol{\omega }}}^{(k)}+{{{\boldsymbol{\varepsilon }}}_{i}}^{(k)},$$

where ***ω***^(*k*)^ is the 10 × *p* coefficient matrix, and ${{{\boldsymbol{\varepsilon }}}_{i}}^{(k)}$ is the vector of random errors. ${\hat{{\boldsymbol{\omega }}}}^{(k)}$, the estimate of ***ω***^(*k*)^, can be obtained using the (regularized) least squares method. With this regression, the levels of CNVs are then decomposed into two components. The first is ${\hat{{\boldsymbol{z}}}}_{i}^{(k)}={{\boldsymbol{x}}}_{pc,i}^{(k)}{\hat{{\boldsymbol{\omega }}}}^{(k)}$, which, loosely speaking, contains information in CNV that overlaps with that in gene expression. The second component is ${{\boldsymbol{z}}}_{i}^{(k)}-{\hat{{\boldsymbol{z}}}}_{i}^{(k)}$, which contains independent information of CNV. As opposed to use the original CNV measurements in regression, only the second component is used. Similar to in the first step, we select the top ten sparse PCs of ${{\boldsymbol{z}}}_{i}^{(k)}-{\hat{{\boldsymbol{z}}}}_{i}^{(k)}$ with the largest variances, which are denoted as ${{\boldsymbol{z}}}_{pc,i}^{(k)}=({z}_{pc,i1}^{(k)},\ldots ,{z}_{pc,i10}^{(k)})$, $i=1,\ldots ,\,{n}^{(k)}$.

For *k* = 1, 2, representing bipolar disorder and schizophrenia, consider the logistic regression model

$$P({y}_{i}^{(k)}=1)=\frac{1}{1+\exp (-({{\boldsymbol{x}}}_{pc,i}^{(k)}{{\boldsymbol{\theta }}}_{x}^{(k)}+{{\boldsymbol{z}}}_{pc,i}^{(k)}{\theta }_{z}^{(k)}+{\alpha }^{(k)}))},$$

with ${{\boldsymbol{\theta }}}_{x}^{(k)}=({\theta }_{x1}^{(k)},\ldots ,{\theta }_{x,10}^{(k)})^{\prime}$ and ${{\boldsymbol{\theta }}}_{z}^{(k)}=({\theta }_{z1}^{(k)},\mathrm{...},{\theta }_{z,10}^{(k)})^{\prime}$ being the vectors of regression coefficients, and *α*^(*k*)^ being the intercept. The unknown regression coefficients are then estimated using the standard maximum likelihood approach.

#### Rationale

The above analysis has been motivated by the following considerations. (1) In the analysis of omics data, high dimensionality and strong correlation are not uncommon. SPCA is adopted in the first step, which can effectively tackle both problems. SPCA applies penalization to the loadings of PCs, which leads to the loadings of unimportant variables estimated as exactly zero. As such, sparse PCs are linear combinations of selected important variables with nonzero loadings, and only these important variables can enter the logistic regression model. SPCA is superior to the “standard” PCA by removing “noises” and “focusing” more on important variables, leading to more interpretable results. Here SPCA is conducted on gene expression, which is “closer” to disease outcome than CNV. In our analysis, the number of sparse PCs is fixed as ten, which leads to low computational cost and satisfactory numerical performance. We note that this choice may be slightly subjective, and there are other ways to determine the number of sparse PCs. For example, it can be selected using the cumulative variance contribution rate, following the traditional principal component analysis. It can also be selected based on model selection techniques, such as the Bayesian Information Criterion (BIC) and cross validation. However, in this set of analysis, the main goal is to construct an outcome model as opposed to selecting significant PCs. As such, we fix the number of PCs but also note that this may need adjustment in other studies. In addition, as suggested in the literature^12^, SPCA can be replaced by other dimension reduction techniques, for example, partial least squares. (2) In a “standard” regulation analysis, gene expressions are regressed on CNVs, which, along with other regulators, regulate gene expressions. However, in the second step of our analysis, our goal is to remove “redundant information” in CNVs (that overlaps with that in gene expressions). As such, a “reversed regression” of CNVs on gene expressions is proposed. With the low dimensionality of the sparse PCs of gene expressions, this step of regression can be easily realized. The strategy of decomposition has also been considered in the literature^32^, however, under different settings and using different techniques. We note that there are more complex techniques for extracting overlapping information, for example, based on nonlinear modeling. However, such analysis can be challenged by the high dimensionality and low sample size as in this dataset. Linear regression has been adopted in the published gene expression-CNV (and other regulators) association studies^33,34^ and shown to be effective. As the main goal here is outcome model building as opposed to modeling the CNV-gene expression relationships, it is sensible to adopt not overly complex methods (which may not be “perfect” for overlapping information extraction). (3) The third step of analysis is a “standard” regression, where we collectively analyze gene expression and its regulator CNV to more comprehensively and more informatively describe disease outcome. Published studies have suggested that multiple types of omics changes, including gene expressions, CNVs, DNA methylations, and others, are potentially associated with disease outcomes^35^. Different types of omics measurements are interconnected. As such they may have overlapping information. On the other hand, it is also suggested that they can have independent information. Here, we use the “residual” ${{\boldsymbol{z}}}_{i}^{(k)}-{\hat{{\boldsymbol{z}}}}_{i}^{(k)}$ to describe the information of CNVs that is independent from gene expressions and potentially has direct effects on disease outcomes not captured by gene expressions^36^. Here we note that ${{\boldsymbol{z}}}_{i}^{(k)}-{\hat{{\boldsymbol{z}}}}_{i}^{(k)}$ is not random error in “standard” regression analysis. Rather it may contain (potentially important) information in CNV that is not reflected in gene expression. Including it in analysis makes the proposed model significantly different from the gene-expression-only analysis. The logistic model can be replaced by other models depending on data/model settings.

### Horizontal integrative analysis for disease marker identification

In this analysis, the goal is to identify omics markers that are associated with diseases. With the relatedness of bipolar disorder and schizophrenia, integrative analysis is conducted to borrow information across diseases so as to generate more reliable marker identification and estimation. The penalization technique is adopted to accommodate high data dimensionality as well as select relevant markers. Further an additional penalty is introduced to facilitate borrowing information.

The same analysis can be conducted on different types of omics measurements separately. To avoid confusion, we take gene expression as an example. For *k* = 1, 2, representing bipolar disorder and schizophrenia respectively, consider the logistic regression model

$$P({y}_{i}^{(k)}=1)=\frac{1}{1+\exp (-({{\boldsymbol{x}}}_{i}^{(k)}{{\boldsymbol{\beta }}}^{(k)}+{\alpha }^{(k)}))},$$

where ${{\boldsymbol{\beta }}}^{(k)}=({\beta }_{1}^{(k)},\mathrm{...},{\beta }_{p}^{(k)})^{\prime}$is the *p*-dimensional coefficient vector, and *α*^(*k*)^ is the unknown intercept. To select important gene expressions, and to accommodate high data dimensionality, consider the penalized estimation with objective function

1 $$-l({{\boldsymbol{\beta }}}^{(1)})-l({{\boldsymbol{\beta }}}^{(2)})+{\lambda }_{1}{\sum }_{k=1}^{2}{\sum }_{j=1}^{p}|{\beta }_{j}^{(k)}|+\frac{{\lambda }_{2}}{2}\rho ({{\boldsymbol{\beta }}}^{(1)},{{\boldsymbol{\beta }}}^{(2)}),$$

where *l*(***β***^(*k*)^) is the log-likelihood function, and *λ*_1_ > 0 and *λ*_2_ > 0 are the tuning parameters. Here it is noted that we have suppressed the dependence on the intercepts, which are low-dimensional and not subject to penalization. With respect to *ρ*(***β***^(1)^, ***β***^(2)^), we consider two proposals, which have been considered in the literature but under quite different settings. The first is the *magnitude-based shrinkage penalty*

2 $${\sum }_{j=1}^{p}{\sum }_{k\ne k^{\prime} }{({\beta }_{j}^{(k)}-{s}_{j}^{(kk^{\prime} )}{\beta }_{j}^{(k^{\prime} )})}^{2},$$

where ${s}_{j}^{(kk^{\prime} )}={\rm{I}}\{{\rm{Sgn}}({\beta }_{j}^{(k)})={\rm{Sgn}}({\beta }_{j}^{(k^{\prime} )})\}$ with Sgn(·) and I(·) being the sign and indicator functions. The second is the *sign-based shrinkage penalty*

3 $${\sum }_{j=1}^{p}{\sum }_{k\ne k^{\prime} }{({\rm{Sgn}}({\beta }_{j}^{(k)})-{\rm{Sgn}}({\beta }_{j}^{(k^{\prime} )}))}^{2}.$$

The estimate is defined as the minimizer of (1). The nonzero components of ***β***^(*k*)^ correspond to important variables that will be concluded as associated with the disease risk. The proposed integrative analysis for disease marker identification with penalties (2) and (3) are referred to as B1 and B2, respectively.

#### Rationale

Joint analysis, which can accommodate the combined effects of multiple genes in a single model, is conducted. Compared to marginal analysis that analyzes one gene at a time and has a risk of missing factors with weak marginal but strong joint effects, the proposed analysis can more effectively describe disease biology, that is, outcomes and phenotypes of complex diseases are usually associated with the joint effects of multiple gene expression anomalies. In objective function (1), if *λ*_2_ = 0, then the analysis simplifies to two Lasso estimations, with one for each disease. Here Lasso can be replaced by other sparse penalties as well as other techniques that can conduct regularized estimation and variable selection. The key advancement is the $\frac{{\lambda }_{2}}{2}\rho ({{\boldsymbol{\beta }}}^{(1)},{{\boldsymbol{\beta }}}^{(2)})$ penalty term. Intuitively, with the relatedness of the two diseases, this newly added penalty promotes certain similarity between the models for the two diseases, thus realizing information borrowing. More specifically, the magnitude-based shrinkage penalty (2) promotes the magnitudes of the two sets of omics effects to be similar, that is, *quantitative* similarity. More specifically, in (2), for each gene, if the signs of the corresponding coefficients for the two diseases are the same, then the value difference between the two coefficients is shrunk toward zero. In contrast, the sign-based penalty (3) promotes the same signs, that is, *qualitative* similarity. In (3), for each gene, the sign difference between two coefficients for the two diseases is shrunk towards zero. As such, the two important sets identified under the magnitude-based shrinkage penalty may tend to have more similar effect magnitudes, while those under the sign-based shrinkage penalty may tend to have more overlaps. In a sense, the former one promotes stronger similarity than the latter one. Although in the literature the relatedness of bipolar disorder and schizophrenia has been suggested, it is not clear “how similar” they are. As such, it is prudent to examine both penalties. In addition, in future analysis when the relatedness of other diseases is less clear, both penalties can be useful.

### Horizontal integrative analysis of gene expression-CNV regulations

For many complex diseases including mental disorders, the dysregulation of omics measurements (for example, gene expressions by CNVs) is an important cause of disease development. In this analysis, we examine the regulations of gene expressions by CNVs. It is noted that both sides of the regression are high dimensional, making the analysis particularly challenging. As such, it can be more important to borrow information across diseases. The integration strategy taken here is consistent with that in the above analysis.

Specifically, for bipolar and schizophrenia samples, consider the regression model

4 $${x}_{ij}^{(k)}={{\boldsymbol{z}}}_{i}^{(k)}{{\boldsymbol{\eta }}}_{j}^{(k)}+{\delta }_{ij}^{(k)},\,j=1,\ldots ,p,$$

where ${{\boldsymbol{\eta }}}_{j}^{(k)}=({\eta }_{j1}^{(k)},\ldots ,{\eta }_{jp}^{(k)})^{\prime}$ is the *p*-dimensional coefficient vector, and ${\delta }_{ij}^{(k)}$ is the random error. For estimating ${{\boldsymbol{\eta }}}_{j}^{(k)}$’s, we consider the objective function

5 $${\sum }_{k=1}^{2}\frac{1}{2{n}^{(k)}}{\sum }_{j=1}^{p}{\sum }_{i=1}^{{n}^{(k)}}{({x}_{ij}^{(k)}-{{\boldsymbol{z}}}_{i}^{(k)}{{\boldsymbol{\eta }}}_{j}^{(k)})}^{2}+{\lambda }_{3}{\sum }_{k=1}^{2}{\sum }_{j=1}^{p}{\sum }_{l=1}^{p}|{\eta }_{jl}^{(k)}|+{\lambda }_{4}{\sum }_{j=1}^{p}\rho ({{\boldsymbol{\eta }}}_{j}^{(1)},{{\boldsymbol{\eta }}}_{j}^{(2)})$$

where *λ*_3_ > 0 and *λ*_4_ > 0 are the tuning parameters, and $\rho ({{\boldsymbol{\eta }}}_{j}^{(1)},{{\boldsymbol{\eta }}}_{j}^{(2)})$ is defined similarly as in (2) and (3), promoting similarity in magnitudes and signs of the estimates. In (5), we conduct joint analysis which fits all CNVs in one model. With penalization, the estimated coefficients of some CNVs can be exactly zero. A nonzero component of ${{\boldsymbol{\eta }}}_{j}^{(k)}$ corresponds to a regulation between the corresponding gene expression and CNV, with the magnitude and sign describing the strength and “direction” of regulation. The proposed integrative analysis of gene expression-CNV regulations with penalties similar to those in (2) and (3) are referred to as C1 and C2, respectively.

#### Rationale

When the sample size is limited but the numbers of gene expressions and CNVs are large, we describe regulations using linear regression models. The Lasso penalization technique is adopted to accommodate high dimensionality and, more importantly, identify regulations. Similar to that in Section 2.4, the magnitude and sign based penalties are imposed to facilitate information borrowing and promote similarity between the two closely related diseases. The proposed penalized objective function (5) has been motivated by the considerations that most components of ${{\boldsymbol{\eta }}}_{j}^{(k)}$’s are zero, and ${{\boldsymbol{\eta }}}_{j}^{(1)}$ and ${{\boldsymbol{\eta }}}_{j}^{(2)}$ for the two diseases have a certain degree of similarity. It has been suggested that one gene expression is usually regulated by only a small number of CNVs, and one CNV usually affects only a few gene expression levels^34^, leading to the sparsity of ${{\boldsymbol{\eta }}}_{j}^{(k)}$’s. The similarity between the two diseases and hence their regression models has been discussed in Section 1. In a similar spirit, integrative analysis can also be conducted on diseased and normal samples, which may facilitate a better understanding of disease etiology by identifying dysregulations.

### Computation

For the vertical integrative analysis described in Section 2.3, the three steps can be realized using existing software. In particular, in our data analysis, we use R functions *spc*, *lm*, and *glm* for SPCA, linear regression, and logistic regression, respectively. For the two types of horizontal integrative analysis described in Sections 2.4 and 2.5, optimizations cannot be carried out straightforwardly using existing software. In our analysis, they are realized based on the coordinate descent (CD) technique which has been a popular choice in penalization studies and has affordable computational cost. The detailed computational algorithms are described in Appendix. In Table A1 (Appendix), we provide the average computer time, obtained using a regular laptop, for the three types of analysis on simulated data with sample size 10,000. Various values of dimension have been examined. It is observed that the proposed analyses are computationally feasible even for data with a much larger sample size. For example, for the simulated data with *n* = 10,000 and *p* = 500, the proposed horizontal integrative analysis for disease marker identification B1 and B2 takes about 9.3 and 10.9 minutes. To facilitate data analysis and applications beyond this study, we have developed R code and made it publicly available at https://github.com/shuanggema/VHintegr. It can be easily implemented and modified if needed. It is noted that computational cost can be much reduced with parallel computing and more powerful computers.

## Results

### Vertical integrative analysis of multi-omics data

Beyond the integrative analysis method described in Section 2.3 (referred to as A1), we also consider the following alternatives: Approach A2 conducts SPCA with gene expressions and CNVs separately and then stack the top ten PCs together for downstream analysis. This approach uses both gene expression and CNV data, however, there is a lack of attention to the overlapping information. Approaches A3 and A4 use the top ten PCs of gene expressions and CNVs, respectively, and there is a lack of data integration. We conduct analysis on bipolar disorder and schizophrenia, respectively.

We first examine the sparse PCs, which are the building blocks of the outcome models. The numbers of important genes with nonzero PC loadings are provided in Table 1, along with the overlaps between different approach. Detailed estimation results of the PC loadings are provided in Table S1 (Supplementary Excel file). It is observed that the set of important genes under A1 differs from the alternatives. Preliminary literature search suggests that the genes with nonzero loadings under A1 may have important biological implications. For example, gene MAPK1, which has a nonzero loading in the eighth PC for bipolar disorder, has been shown to have an impact on monoamines-related pathways and dendrites development^37^ and have associations with bipolar disorder^37,38^. For bipolar disorder, gene YWHAE has nonzero loadings in the first and tenth PCs. It has been reported in Jie, *et al*.^39^, which investigated its 11 SNPs, that it plays a critical role in bipolar disorder. Gene TPH1 is identified as important for both bipolar disorder and schizophrenia. It has been suggested as a schizophrenia risk-associated gene^40^. The corresponding tryptophan hydroxylase (TPH) has been proposed as a rate-limiting enzyme that can limit the rate of the synthesis of 5-HT which is implicated in the pathophysiology of schizophrenia^41^. It has also been found that the polymorphisms of rs1800532 and rs1799913 of TPH1 are associated with bipolar disorder^42^. Gene NGF has a nonzero loading in the first PC for bipolar disorder. Published studies have shown that Serum NGF level has a compensatory role of neuroprotection and is significantly correlated with the duration of bipolar disorder^43^. The genetic variants and protein expressions of AKT1 have been investigated for both bipolar disorder and schizophrenia in Karege, *et al*.^44^, which established its critical role. We note that the goal of this analysis is not to identify disease associated genes. However, the above plausible biological interpretations may still provide some support to the proposed analysis.

**Table 1:** Vertical integrative analysis of multi-omics data: number of genes identified by different approaches and their overlaps.

	Bipolar disorder				Schizophrenia			
	A1	A2	A3	A4	A1	A2	A3	A4
A1	67	54	33	36	62	62	33	34
A2		139	33	121		113	33	85
A3			33	15			33	5
A4				121				85

Detailed estimated coefficients for the PCs under different approaches are provided in Table S2 (Supplementary Excel file), which reveals that different approaches lead to different estimations. It is difficult to objectively evaluate which set of estimation is “biologically more meaningful”. As in published studies, we conduct a resampling based prediction evaluation, which can provide some support to the validity of estimation. Specifically, data is randomly split into a training and a testing set. Estimation is generated using the training set and used to make prediction for the testing set subjects. The corrected prediction ratio (CPR) is used to evaluate prediction, where a larger value indicates a better prediction. The procedure is repeated 200 times, and the summary results are provided in Table 2. It is observed that all approaches have moderate CPRs, which are likely caused by the small sample sizes and low signal levels. In addition, both bipolar and schizophrenia have a large number of other risk factors. In this analysis, only omics variables are considered. Taking both diseases into consideration, integration analysis A1 has a small advantage over the alternatives.

**Table 2:** Vertical integrative analysis of multi-omics data: prediction performance of different approaches, mean (sd) of CPR.

Approach	Bipolar disorder	Schizophrenia
_A1_	0.50 (0.15)	0.53 (0.14)
_A2_	0.46 (0.14)	0.48 (0.13)
_A3_	0.57 (0.14)	0.47 (0.16)
_A4_	0.39 (0.13)	0.51 (0.14)

### Horizontal integrative analysis for disease marker identification

Besides the integrative analysis methods described in Section 2.4 with penalties (2) (referred to as B1) and (3) (referred to as B2), we also consider an alternative analysis, referred to as B3, which analyzes the two diseases separately and applies Lasso to accommodate high dimensionality and select relevant markers. This comparison can straightforwardly show the merit of horizontal integration. Detailed estimation results are provided in Table S3 (Supplementary Excel file). The numbers of gene expressions identified by different approaches and their overlaps are provided in Table 3, which suggests that different approaches lead to different findings. In addition, there are also variations across pathways and diseases. To better comprehend the adopted similarity-based penalties (2) and (3), we further provide the overlaps of the identified genes between the two diseases in Table 4. Overall, compared to B3, B1 and B2 are observed to identify more overlapping gene expressions associated with both diseases. For example, for the first pathway, the numbers of overlapping gene expressions are 35 (B1), 32 (B2), and 4 (B3). With the close relatedness of bipolar and schizophrenia, the integrative analysis results can be sensible. For the gene expressions identified by B1 and B2, literature search suggests that they may have important biological implications. For example, gene BRCA1 in the ubiquitin mediated proteolysis pathway, with nonzero coefficients for both diseases, is relevant to cell cycle. It has been reported to be down-regulated in schizophrenia patients^45^, supporting BRCA1 as a potential biomarker for schizophrenia. Regarding to gene Bax in the tryptophan metabolism pathway, Bax/Bcl-2 ratio has been found to be 50% higher in schizophrenia patients than nonpsychiatric comparison subjects^46^. Gene PIK3CA encodes the alpha catalytic subunit of the PI3K enzyme, of which the aberrant signaling has been identified as a factor in the pathophysiology of multiple psychiatric disorders including schizophrenia and autism^6^. Published studies have shown that the expression of gene Bcl-2 can impact intracellular calcium (Ca2+) homeostasis (ICH) in bipolar disorder^47^. In addition, in the neurotrophin signaling pathway, it has been reported that the expression of gene PSEN1 is significantly decreased in schizophrenia and bipolar patient groups, supporting PSEN1 as a potential biomarker for both diseases^48^.

**Table 3:** Horizontal integrative analysis for disease marker identification: numbers of gene expressions identified by different approaches and their overlaps.

Pathway	Approach	Bipolar disorder			Schizophrenia		
		B1	B2	B3	B1	B2	B3
1	B1	62	25	11	68	18	8
	B2		32	6		32	5
	B3			12			13
2	B1	14	9	4	16	9	1
	B2		24	4		19	3
	B3			4			3
3	B1	27	10	6	12	7	1
	B2		33	8		34	8
	B3			18			11

**Table 4:** Horizontal integrative analysis for disease marker identification: overlaps of the identified gene expressions between two diseases with different approaches.

Pathway	Approach	Bipolar disorder	Schizophrenia	Overlaps
1	B1	62	68	35
	B2	32	32	32
	B3	12	13	4
2	B1	14	16	10
	B2	24	19	15
	B3	4	3	1
3	B1	27	12	4
	B2	33	34	27
	B3	18	11	4

#### Simulation

To better comprehend the operating characteristics of the two types of shrinkage penalties, we conduct simulation under various scenarios to evaluate their identification performance. The specific settings are as follows. First, set *n*^(*k*)^ = 40, *k* = 1, 2, 3, and *p* = 100. Then, following the literature for example the human disease network studies^49,50^, the similarity between diseases is described using marker similarity, where diseases sharing with more common important markers or having more similar marker effects are indicated to be more similar. Specifically, gene expression values for the *k*th type of mental disorders are generated from a multivariate normal distribution N(***μ***_*k*_,**Σ**)with a mean vector ***μ***_*k*_ = (*μ*_*k*1_, …, *μ*_*kp*_) and a block-diagonal covariance matrix **Σ**, and those for the normal controls are generated from N(0, **Σ**). Here, the first eight variables are set as associated with the disease outcomes with the corresponding elements of ***μ***_*k*_ being nonzero, and the rest of ***μ***_*k*_ are zeros. **Σ** has two blocks corresponding to the important variables and unimportant ones, respectively, where each block has an auto-regressive structure with the correlation coefficient between the *j*th and *k*th variables being 0.3^|*j*−*k*|^. Set ***μ***_1_ = (−1, −1, −1, −1, 2, 2, 2, 2, 0, …, 0). We consider various values of ***μ***_2_ to generate different levels of disease similarity. Scenario I has ***μ***_1_ = ***μ***_2_, that is, with the same signs and same magnitudes. Here two diseases have strong similarity with the same markers as well as the same association effects. Scenarios II and III have ***μ***_1_ and ***μ***_2_ with the same signs but magnitudes having certain differences. In particular, ***μ***_2_ = (−1, −1, −4, −4, 2, 2, 4, 4, 0, …, 0) for scenario II, and ***μ***_2_ = (−2, −2, −0.5, −0.5, 3, 3, 1, 1, 0, …, 0) for scenario III. Scenario IV has ***μ***_1_ and ***μ***_2_ with the same nonzero components but various conflicting signs. In particular ***μ***_2_ = (2, 2, −0.5, −0.5, 3, 3, −1, −1, 0, …, 0). It is noted that generating variables with normal distributions for two diseases directly corresponds to logistic regression models. We acknowledge that the simulation settings may be considerably simpler than practical data. However, such settings have been extensively adopted in the literature and are sufficient for demonstrating the proposed analysis.

For the four scenarios, we compute the average TPR (true positive rate) and FPR (false positive rate) values over 100 replicates in Table A2 (Appendix) to evaluate identification performance. It is observed that the proposed B1 and B2 perform much better than B3, with larger TPR and smaller FPR values under Scenarios I-III. Under Scenario IV, where the sign and magnitude consistency do not hold, B1 and B2 still have performance comparable to B3. Compared to B2, B1 has superior performance under Scenarios I and II, and inferior performance under Scenarios III and IV, suggesting that the magnitude-based penalty may be favorable when two diseases have stronger similarity. This simulation also suggests that for practical data analysis, where the level of similarity is unknown, it is worthwhile to have both approaches.

### Horizontal integrative analysis of gene expression-CNV regulations

Besides the integrative analysis methods described in Section 2.5 with penalties similar to those in (2) (referred to as C1) and (3) (referred to as C2), we also consider an alternative for a direct comparison. The approach C3 analyzes the gene expression-CNV regulation for the two diseases separately using the Lasso approach for accommodating high dimensionality and conducting variable selection. The detailed estimation results are provided in Tables S4–S6 (Supplementary Excel file), and the summary is provided in Table 5. It is observed that integrative analysis C1 and C2 generate findings different from the benchmark C3, with different sets of nonzero coefficients and hence concluding different gene expression-CNV regulations. Such differences are sensible, as the three approaches have fundamentally different strategies, with C1 and C2 promoting similarity in magnitudes and signs respectively, and C3 paying no attention to the potential similarity.

**Table 5:** Horizontal integrative analysis of gene expression-CNV regulations: numbers of regulations identified by different approaches and their overlaps.

Pathway		Bipolar disorder			Schizophrenia		
		C1	C2	C3	C1	C2	C3
1	C1	197	23	3	279	31	19
	C2		203	2		230	30
	C3			281			590
2	C1	49	4	11	54	9	2
	C2		40	1		46	3
	C3			117			110
3	C1	242	13	9	240	18	12
	C2		147	2		147	8
	C3			214			631

Besides identification, we also more closely examine the estimation results. Specifically, we compute the differences of the estimated coefficient matrices for any two of bipolar, schizophrenia, and normal. Here, the gene expression-CNV regulations for the normal are estimated using Lasso separately. Two distances are considered. The first is defined as ${\rm{Dist}}({{\boldsymbol{\eta }}}_{j}^{(k)},{{\boldsymbol{\eta }}}_{j}^{(k^{\prime} )})=\mathop{\sum }\limits_{l=1}^{p}{({{\boldsymbol{\eta }}}_{jl}^{(k)}-{{\boldsymbol{\eta }}}_{jl}^{(k^{\prime} )})}^{2},\,k\ne k^{\prime}$, which takes into account both magnitude and sign; and the second is defined as ${\rm{SignDist}}({{\boldsymbol{\eta }}}_{j}^{(k)},{{\boldsymbol{\eta }}}_{j}^{(k^{\prime} )})={\sum }_{l=1}^{p}{({\rm{Sgn}}({{\boldsymbol{\eta }}}_{jl}^{(k)})-{\rm{Sgn}}({{\boldsymbol{\eta }}}_{jl}^{(k^{\prime} )}))}^{2},\,k\ne k^{\prime}$, which focuses on the “directions” (signs) of regulations. Results are shown in Table 6. It is observed that, under C1 and C2, both measures between two diseases are significantly smaller than those between disease and normal. It is expected that the similarity between diseases is higher than that between disease and normal. As such, the resulted distances are sensible, which can provide support to the validity of integration. A closer examination of the identified gene expression-CNV regulations is also taken, which suggests that the identified regulations are biologically sensible. Of note in the proposed analysis, both cis- and trans-acting regulations are considered. As a representative example, we consider gene ATF4. Disruption in schizophrenia 1 (DISC1) and its molecular cascade have an influence on the pathophysiology of schizophrenia and bipolar disorder. ATF4 can encode proteins that interact with DISC1, suggesting its important role in the pathophysiology of the two diseases^51^. With approaches C1 and C2, the expression of gene ATF4 is identified to be regulated by three CNVs (PLCG2, PTPN11, CALML5) besides the cis-acting CNV. In contrast, the alternative C3 misses these regulations.

**Table 6:** Horizontal integrative analysis of gene expression-CNV regulations: differences between coefficient matrices. BD, SZ, and N stand for bipolar disorder, schizophrenia, and normal.

Pathway	Approach	SZ-N	BD-N	BD-SZ
$${\bf{Dist}}({{\boldsymbol{\eta }}}_{{\boldsymbol{j}}}^{({\boldsymbol{k}})},{{\boldsymbol{\eta }}}_{{\boldsymbol{j}}}^{({\boldsymbol{k}}^{\prime} )})$$				
1	C1	226.21	223.84	137.90
	C2	241.35	243.60	191.94
	C3	300.31	262.70	281.58
2	C1	172.89	159.21	73.44
	C2	157.86	153.72	72.05
	C3	213.43	180.20	189.58
3	C1	216.90	209.61	134.28
	C2	213.80	207.03	132.57
	C3	499.10	206.39	468.94
$${\bf{SignDist}}({{\boldsymbol{\eta }}}_{{\boldsymbol{j}}}^{({\boldsymbol{k}})},{{\boldsymbol{\eta }}}_{{\boldsymbol{j}}}^{({\boldsymbol{k}}^{\prime} )})$$				
1	C1	30.46	29.05	21.77
	C2	29.61	29.22	20.71
	C3	35.54	30.98	29.68
2	C1	15.13	14.70	9.95
	C2	14.87	14.46	9.17
	C3	16.64	16.73	15.46
3	C1	30.63	30.40	21.40
	C2	28.79	28.83	17.49
	C3	37.00	29.70	28.83

#### Simulation

To obtain further insights into the identification performance of our horizontal integrative analysis, we conduct simulation based on real data. Specifically, we use the observed CNV measurements as predictors and resampling to generate desirable variations across multiple simulation replicates. Each gene expression is regulated by 10% of the CNV measurements, where a half of the corresponding nonzero regression coefficients in (4) are randomly generated from Uniform(0.6, 1.2), and the other half are randomly generated from Uniform(−1.2, −0.6). In addition, we reinforce that, for each gene expression, bipolar disorder and schizophrenia share six important CNVs, and the corresponding coefficients have the same signs. Random errors are generated from N(0, 1), and gene expression values are computed from linear regression models. To evaluate identification, TPR and FPR values are computed. The averages computed based on 100 replicates are provided in Table A3 (Appendix). The proposed two approaches are observed to have better performance for all the three pathways. They can identify the majority of true gene expression-CNV associations with a low FPR. For example, for the first pathway of bipolar disorder, C1 and C2 have (TPR, FPR) = (0.982, 0.019) and (0.992, 0.011), compared to (0.843, 0.046) for C3. This provides support to the validity of the proposed horizontal integrative analysis.

## Discussion

In mental disorder research, omics profiling is getting routine. Two major objectives of omics profiling studies, as shown in the literature, are to construct outcome models using omics measurements and to identify disease associated risk factors. In addition, for almost all diseases including mental disorders as well as normal subjects, it is of interest to understand the regulations among different types of omics measurements. In this sense, this study has not proposed new analysis goals. Rather, the goal of integrative analysis is to better achieve those goals via integrating data on multiple related diseases and on multiple types of omics measurements. In recent biomedical studies, meta-analysis has played a critical role in summarizing results from multiple Genome Wide Association and other types of studies. Different from the summary results-based strategy, the proposed integrative analysis introduces integration of original data in the discovery process. Published studies, for example, Li, *et al*.^52^, Shen, *et al*.^53^, and Zhang and Zhang^54^, have shown that integrative analysis can more effectively integrate information and improve analysis results. As demonstrated by data analysis in this article, integrative analysis is highly feasible and practical with mental disorders. Integrative analysis has been highly successful, for example in cancer research. It is reasonable to expect similar successes in mental disorder research in the near future. This article may provide a timely showcase of integrative analysis with mental disorders.

Our study introduces three types of integrative analysis with different strategies. The first type integrates multi-omics data for a single type of disease. The second type integrates multiple related diseases with one type of omics data. The third type integrates multiple related diseases for identifying regulations between two types of omics data. Researchers may be able to conduct one type or multiple types of the proposed analysis according to the available data. For example, with SNP data from the Psychiatric Genomics Consortium (PGC) on nine types of psychiatric disorders, researchers can conduct the second type of integrative analysis for disease marker identification. With eQTL studies, researchers can conduct the third type of integrative analysis to analyze gene expression-SNP relationships if data are available on different types of mental disorders. In addition, with the development of large-scale genomic projects, there are an increasing number of studies that have collected both disease and omics data. For example, Pai, *et al*.^55^ performed a multi-omics study of neurons isolated from bipolar disorder, schizophrenia, and normal patients, where both DNA methylations and gene expressions were measured. Data are publicly available at the GEO website with GEO accession GSE112525. Multi-omics data have also been collected for other types of related diseases, such as type II diabetes and obesity^56^, obese, bland steatosis and early non-alcoholic fatty liver disease^57^, and others. As mental disorders have been posing an increasing public health concern, it is expected that samples with both disease and omics data for mental disorders will be accumulated at a fast pace in the near future, enabling broader applications of our approaches.

It is noted that integrative analysis methodologies are under fast development, and what has been conducted in this article is only a small subset of integrative analysis. There can be many other possibilities, for example conducting simultaneous vertical and horizontal integrations, accommodating more types of omics measurements, and conducting integrative analysis based on high-dimensional techniques other than penalization. The analysis conducted in this article can be potentially improved in multiple aspects. For mental disorders, clinical, environmental, socioeconomic, and other risk factors may also play critical roles. With limitations in data, such confounders are not included in analysis. When available, they can be easily incorporated in modeling. For example, a more comprehensive outcome model may consist of “confounders + omics measurements”. Confounders usually have a low dimension and are pre-selected. Their coefficients will not be subject to penalized selection. The adopted techniques are based on penalization, which determines whether an effect is important by examining whether its coefficient is nonzero. In “classic” low-dimensional statistical modeling, inference is the popular tool for determining significance. High-dimensional inference with penalization is extremely challenging and still an open problem. It is beyond the scope of this article to develop inference techniques for penalized integrative analysis. Our data analysis has generated some sensible findings that are different from the benchmark alternatives. The analyzed data are limited in multiple aspects, including for example a small sample size, limited omics measurements, and limited outcome variables. As such, the findings may need to be taken with cautious. Ultimately, functional/experimental validations and additional independent data will be needed to confirm our findings. However, our data analysis can already demonstrate the implementation and effectiveness of integrative analysis to a great extent. Overall, the integrative analysis techniques and data analysis results are expected to be useful for mental disorder research.

## Supplementary information

Supplementary materials

Table S1

Table S2

Table S3

Table S4

Table S5

Table S6
