# Supplementary material for: Horizontal and vertical integrative analysis methods for mental disorders omics data
Source: Sci Rep. 2019 Sep 17;9:13430. doi: 10.1038/s41598-019-49718-5 (PMC6748966; doi:10.1038/s41598-019-49718-5)
Supplement: Supplementary file 1 — Supplementary materials [file 41598_2019_49718_MOESM1_ESM.docx]

**Horizontal and vertical integrative analysis methods for mental disorders omics data**

Shuaichao Wang^1^, Xingjie Shi^2^, Mengyun Wu^3*^, Shuangge Ma^4*^

^1^ SJTU-Yale Joint Center for Biostatistics, Department of Bioinformatics and Biostatistics, School of Life Sciences and Biotechnology, Shanghai Jiao Tong University, Shanghai 200240, China

^2^School of Economics, Nanjing University of Finance and Economics, Nanjing 210046, China

^3^School of Statistics and Management, Shanghai University of Finance and Economics, Shanghai 200433, China

^4^Department of Biostatistics, Yale University, New Haven, CT 06520, USA

Correspondence:

*Mengyun Wu, School of Statistics and Management, Shanghai University of Finance and Economics, Shanghai, China.

*Shuangge Ma, Department of Biostatistics, Yale University, New Haven, CT, USA

Email: wu.mengyun@mail.shufe.edu.cn or shuangge.ma@yale.edu

**Appendix**

For optimizing the penalized objective functions (1) and (5) in Sections 2.4 and 2.5, we use the coordinate descent (CD) technique, which has been a popular choice in penalization studies. The CD technique optimizes the objective function with respect to one parameter at a time and iteratively cycles through all parameters until convergence is reached.

With fixed tuning parameters, the CD algorithm for the penalized objective function (1) proceeds as follows.

(1) Initialize $t=0$, $\left( \alpha^{(k)} \right)^{(t)}=0$, $\left( \boldsymbol{\beta}^{(k)} \right)^{(t)}=\left( 0,\ldots,0 \right)^{'}, k=1,2$, where $\left( \alpha^{(k)} \right)^{(t)}$ and $\left( \boldsymbol{\beta}^{(k)} \right)^{(t)}$ denote the estimates of $\alpha^{(k)}$ and $\boldsymbol{\beta}^{(k)}$ at iteration $t$.

(2) Compute

$\nabla_{0}l^{(k)}=\sum_{i=1}^{n^{(k)}} \left( y_{i}^{(k)}-\pi_{i}^{(k)} \right)$, $\nabla_{0}^{2}l^{(k)}=\sum_{i=1}^{n^{(k)}} \pi_{i}^{(k)}\left( 1-\pi_{i}^{(k)} \right)$,

where $\pi_{i}^{(k)}$ is the estimated probability for subject $i$under the logistic model using the current estimate.

Update $\left( \alpha^{(k)} \right)^{\left( t+1 \right)}=\left( \alpha^{(k)} \right)^{\left( t \right)}-\frac{\nabla_{0}l^{(k)}}{\nabla_{0}^{2}l^{(k)}}$.

For $k=1, 2$, $j=1,\ldots,p$, carry out the following steps sequentially.

(2.1) Compute

$\nabla_{j}l^{(k)}=\sum_{i=1}^{n^{(k)}} \left( y_{i}^{(k)}-\pi_{i}^{(k)} \right)x_{ij}^{(k)}, {\mathrm{and} \nabla}_{j}^{2}l^{(k)}=-\sum_{i=1}^{n^{(k)}} \pi_{i}^{(k)}\left( 1-\pi_{i}^{(k)} \right)\left( x_{ij}^{(k)} \right)^{2}$.

(2.2) If$\rho\left( \boldsymbol{\beta}^{\left( 1 \right)},\boldsymbol{\beta}^{\left( 2 \right)} \right)$ is the magnitude-based penalty (2), compute

$a=-2\nabla_{j}^{2}l^{\left( k \right)}+\lambda_{2}, \mathrm{and} b=-2\nabla_{j}^{2}l^{(k)}\left( \beta_{j}^{(k)} \right)^{(t)}+2\nabla_{j}l^{(k)}+\lambda_{2}\sum_{k'\neq k} s_{j}^{(kk')}\left( \beta_{j}^{(k')} \right)^{(t)}$.

If $\rho\left( \boldsymbol{\beta}^{\left( 1 \right)},\boldsymbol{\beta}^{\left( 2 \right)} \right)$ is the sign-based penalty (3), compute

$a=-2\nabla_{j}^{2}l^{\left( k \right)}+\frac{\lambda_{2}}{\left( \left( \beta_{j}^{(k)} \right)^{(t)}+\chi\right)^{2}}$,

$b=-2\nabla_{j}^{2}l^{\left( k \right)}\left( \beta_{j}^{\left( k \right)} \right)^{\left( t \right)}+2\nabla_{j}l^{\left( k \right)}+\lambda_{2}\sum_{k^{'}\neq k} \frac{\left( \beta_{j}^{\left( k^{'} \right)} \right)^{\left( t \right)}}{\left( \left( \beta_{j}^{\left( k \right)} \right)^{\left( t \right)}+\chi\right)\left( \left( \beta_{j}^{\left( k^{'} \right)} \right)^{\left( t \right)}+\chi\right)}$,

where $\chi$ is a small positive number, which is set as 0.01 in our numerical study.

(2.3) Update $\left( \beta_{j}^{\left( k \right)} \right)^{\left( t+1 \right)}=\frac{\mathrm{Sgn}(b)}{a}(\left| b \right|-\lambda_{1})_{+}$, where $(\left| b \right|-\lambda_{1})_{+}=\left\{ \begin{aligned} \left| b \right|-\lambda_{1},\mathrm{if} \left| b \right|-\lambda_{1}>0 \\ 0 , \mathrm{otherwise} \end{aligned} \right.$.

(3) Repeat Step (2) until convergence. In our numerical study, convergence is concluded if $\sum_{k=1}^{2} \sum_{j=1}^{p} \left| \left( \beta_{j}^{\left( k \right)} \right)^{\left( t+1 \right)}-\left( \beta_{j}^{\left( k \right)} \right)^{\left( t \right)} \right|<{10}^{-4}$.

The CD algorithm for the penalized objective function (5) proceeds as follows.

1. Initialize $t=0$, $\left( \boldsymbol{\eta}_{j}^{(k)} \right)^{(t)}=\left( 0,\ldots,0 \right)^{'}, k=1, 2, j=1,\ldots,p,$ where $\left( \boldsymbol{\eta}_{j}^{(k)} \right)^{(t)}$ denotes the estimate of $\boldsymbol{\eta}_{j}^{(k)}$ at iteration $t$.
2. For $k=1, 2$, $j=1,\ldots,p, l=1,\ldots,p$, carry out the following steps sequentially.

(2.1) If$\rho\left( {\boldsymbol{\eta}_{j}}^{(1)},{\boldsymbol{\eta}_{j}}^{(2)} \right)$ is the magnitude-based penalty (2), compute

$c=2\lambda_{4}\left( 1+\sum_{k^{'}\neq k} {s_{lj}^{\left( kk^{'} \right)}}^{2} \right)+\sum_{k=1}^{2} \sum_{i=1}^{n^{\left( k \right)}} \frac{{z_{il}^{\left( k \right)}}^{2}}{n^{\left( k \right)}}$,

$$d=\sum_{k=1}^{2} \sum_{i=1}^{n^{\left( k \right)}} \frac{{z_{il}^{\left( k \right)}}}{n^{\left( k \right)}}\left( x_{ij}^{\left( k \right)}-{\sum_{l^{'}\neq l} \left( \eta_{l^{'}j}^{\left( k \right)} \right)^{\left( t \right)}z}_{l^{'}j}^{\left( k \right)} \right)+{4\lambda}_{4}\sum_{k^{'}\neq k} s_{lj}^{\left( kk^{'} \right)}\left( \eta_{lj}^{\left( k' \right)} \right)^{(t)}.$$

If $\rho\left( {\boldsymbol{\eta}_{j}}^{(1)},{\boldsymbol{\eta}_{j}}^{(2)} \right)$ is the sign-based penalty (3), compute

$$c=2\lambda_{4}\left( 1+\sum_{k^{'}\neq k} {s_{lj}^{\left( kk^{'} \right)}}^{2} \right)-\sum_{k^{'}\neq k} \frac{{2\lambda}_{4}\left( \eta_{lj}^{\left( k' \right)} \right)^{(t)}}{\left( \left| \left( \eta_{lj}^{\left( k' \right)} \right)^{(t)} \right|+\chi\right)^{2}},$$

$d=\sum_{k=1}^{2} \sum_{i=1}^{n^{\left( k \right)}} \frac{{z_{il}^{\left( k \right)}}}{n^{\left( k \right)}}\left( x_{ij}^{\left( k \right)}-{\sum_{l^{'}\neq l} \left( \eta_{l^{'}j}^{\left( k \right)} \right)^{\left( t \right)}z}_{l^{'}j}^{\left( k \right)} \right)-{2\lambda}_{4}\sum_{k^{'}\neq k} \frac{\left( \eta_{lj}^{\left( k' \right)} \right)^{(t)}}{\left( \left| \left( \eta_{lj}^{\left( k' \right)} \right)^{(t)} \right|+\chi\right)\left( \left| \left( \eta_{lj}^{\left( k' \right)} \right)^{(t)} \right|+\chi\right)}$,

(2.2) Update $\left( \eta_{lj}^{\left( k \right)} \right)^{(t+1)}=\frac{\mathrm{Sgn}(d)}{c}(\left| d \right|-\lambda_{3})_{+}$.

1. Repeat Step (2) until convergence. In our numerical study, convergence is concluded if $\sum_{k=1}^{2} \sum_{l=1}^{p} \sum_{j=1}^{p} \left| \left( \eta_{lj}^{\left( k \right)} \right)^{\left( t+1 \right)}-\left( \eta_{lj}^{\left( k \right)} \right)^{\left( t \right)} \right|<{10}^{-4}$.

These approaches involve tuning parameters, which are selected using the BIC approach.

**Table A1** Computer time (minutes) of the proposed approaches for simulated data with sample size 10,000

| Dimension | | Approach | | | | |
| --- | --- | --- | --- | --- | --- | --- |
| Gene expression | CNV | A1 | B1 | B2 | C1 | C2 |
| 100 | 100 | 2.0 | 0.5 | 0.6 | 1.4 | 0.6 |
| 300 | 300 | 16.5 | 3.6 | 3.8 | 18.4 | 13.4 |
| 500 | 500 | 48.4 | 9.3 | 10.9 | 240.2 | 61.3 |

**Table A2** Simulation: horizontal integrative analysis for disease marker identification: average TPRs and FPRs.

| Scenario | Approach | Disease 1 | | Disease 2 | | |
| --- | --- | --- | --- | --- | --- | --- |
|  |  | TPR | FPR |  | B1 | B2 |
| I | B1 | 0.785 | 0.065 |  | 0.762 | 0.059 |
|  | B2 | 0.745 | 0.079 |  | 0.735 | 0.077 |
|  | B3 | 0.555 | 0.066 |  | 0.552 | 0.072 |
| II | B1 | 0.775 | 0.065 |  | 0.706 | 0.004 |
|  | B2 | 0.737 | 0.064 |  | 0.643 | 0.008 |
|  | B3 | 0.562 | 0.084 |  | 0.456 | 0.081 |
| III | B1 | 0.661 | 0.024 |  | 0.618 | 0.008 |
|  | B2 | 0.718 | 0.013 |  | 0.631 | 0.002 |
|  | B3 | 0.625 | 0.108 |  | 0.437 | 0.105 |
|  | B1 | 0.715 | 0.023 |  | 0.705 | 0.011 |
| IV | B2 | 0.721 | 0.026 |  | 0.711 | 0.013 |
|  | B3 | 0.724 | 0.089 |  | 0.717 | 0.066 |

**Table A3** Horizontal integrative analysis of gene expression-CNV regulations: average TPRs and FPRs of different approaches for data-based simulation.

| Pathway | Approach | Bipolar disorder | | Schizophrenia | | |
| --- | --- | --- | --- | --- | --- | --- |
|  |  | TPR | FPR |  | TPR | FPR |
| 1 | C1 | 0.982 | 0.019 |  | 0.982 | 0.018 |
|  | C2 | 0.992 | 0.011 |  | 0.992 | 0.011 |
|  | C3 | 0.843 | 0.046 |  | 0.865 | 0.043 |
| 2 | C1 | 0.968 | 0.043 |  | 0.965 | 0.039 |
|  | C2 | 0.973 | 0.034 |  | 0.964 | 0.032 |
|  | C3 | 0.321 | 0.114 |  | 0.353 | 0.130 |
| 3 | C1 | 0.984 | 0.018 |  | 0.983 | 0.018 |
|  | C2 | 0.988 | 0.013 |  | 0.991 | 0.012 |
|  | C3 | 0.835 | 0.049 |  | 0.819 | 0.067 |

Descriptions of Supplementary Tables S1-S6.

**Table S1:** Analysis results for Section 3.1. Three sheets contain the estimates of loadings of the top 10 sparse PCs of gene expressions (GEs), CNVs, and independent information of CNVs from GEs (CNVs-GEs), respectively.

**Table S2:** Analysis results for Section 3.1: estimated coefficients for the PCs in the logistic regression models under Approaches A1-A4.

**Table S3:** Analysis results for Section 3.2: estimated coefficients for the identified GEs under Approaches B1-B3.

**Table S4:** Analysis results for Section 3.3: estimated coefficients for the identified GE-CNV regulations under C1. Columns correspond to GEs, and rows correspond to CNVs.

**Table S5:** Analysis results for Section 3.3: estimated coefficients for the identified GE-CNV regulations under C2. Columns correspond to GEs, and rows correspond to CNVs.

**Table S6:** Analysis results for Section 3.3: estimated coefficients for the identified GE-CNV regulations under C3. Columns correspond to GEs, and rows correspond to CNVs.
